# Alcohol imagery on New Zealand television

**DOI:** 10.1186/1747-597X-2-6

**Published:** 2007-02-01

**Authors:** Rob McGee, Juanita Ketchel, Anthony I Reeder

**Affiliations:** 1 Social and Behavioural Research in Cancer Group, Department of Preventive and Social Medicine, University of Otago Medical School, Box 913 Dunedin, New Zealand

## Abstract

**Background:**

To examine the extent and nature of alcohol imagery on New Zealand (NZ) television, a content analysis of 98 hours of prime-time television programs and advertising was carried out over 7 consecutive days' viewing in June/July 2004. The main outcome measures were number of scenes in programs, trailers and advertisements depicting alcohol imagery; the extent of critical versus neutral and promotional imagery; and the mean number of scenes with alcohol per hour, and characteristics of scenes in which alcohol featured.

**Results:**

There were 648 separate depictions of alcohol imagery across the week, with an average of one scene every nine minutes. Scenes depicting uncritical imagery outnumbered scenes showing possible adverse health consequences of drinking by 12 to 1.

**Conclusion:**

The evidence points to a large amount of alcohol imagery incidental to storylines in programming on NZ television. Alcohol is also used in many advertisements to market non-alcohol goods and services. More attention needs to be paid to the extent of alcohol imagery on television from the industry, the government and public health practitioners. Health education with young people could raise critical awareness of the way alcohol imagery is presented on television.

## Background

Alcohol imagery is widely shown in a variety of visual media including children's animated films [1], music videos [2], reality television [3], televised sports [4] and advertising [5]. There is a growing evidence to suggest that the advertising of alcohol (especially aggressive advertising of beer) is associated with higher levels of drinking. For example, in a longitudinal cohort study set in Dunedin NZ, boys who were able to recall more advertisements for beer at age 15 years, reported drinking more beer when assessed three years later at age 18 years [6]. This was the first longitudinal demonstration of a predictive relationship between alcohol advertising on television (TV) and later consumption. More recent US research also suggests that alcohol advertising on TV, in magazines and in stores and concerts, predicts subsequent drinking among adolescents exposed to them [7]. The mechanisms through which advertising has these effects remain unclear. Alcohol imagery might play a role in modelling drinking behavior among young people, including encouraging the "normalization" of drinking and intoxication [8]. In a study of Norwegian adolescents, for example, higher levels of exposure to television containing incidental portrayals of alcohol were associated with the development of normative beliefs that drinking is a usual teenage behavior, especially among those who reported having no close friends who drank alcohol [9].

Recently, we examined depictions of tobacco use on NZ television in 2002 and 2004 [10]. One in four programs during prime-time viewing contained tobacco imagery, most of which was "uncritical" of tobacco use. There were about two scenes containing such imagery every hour, a level unchanged since the late 1990s. While the legislative framework in NZ around tobacco has led to greater restrictions on advertising and the sale of tobacco, the reverse is true for alcohol with progressive liberalization of advertising over the last 20 years [5]. We are unaware of any research documenting the amount of alcohol advertising on NZ TV, and the amount of alcohol imagery in programming. This was the topic of our study.

## Methods

In NZ the main free-to-air national channels are TV1 and TV2 (state owned) and TV3 (privately owned). For seven consecutive days in 2004, Friday 25^th ^June to Thursday July 1^st^, we video recorded 98 hours of programs (including movies) shown from 6.00 to 9.30 pm on TV1, TV2 and TV3, as well as C4, a music video channel aimed at 15–29 year olds [11]. There are clearly several different ways of sampling material for content analysis [12]. Our study was restricted in terms of extended access to videoing equipment and other constraints that precluded sampling over longer periods of time. Consequently, we chose 7 consecutive days of prime-time broadcasting following two earlier NZ studies which had examined levels of tobacco imagery in the 1990s [10]. The main proviso we had was that the week chosen was what was usual for TV programming. For example, there were no major sporting events (e.g. Olympic games) or other major events that might take up large amounts of TV program time. It is difficult to determine whether this sampling procedure introduced any bias. As far as we could tell, the week chosen was not out of the ordinary compared with previous and subsequent weeks programming.

A prime interest was to identify scenes that contained alcohol imagery. A scene was identified as a discrete set of camera shots relating to a set piece of "action" within a program, advertisement or trailer, with termination usually indicated by the camera cutting away to a new location, new actors or new staged action piece. As we note elsewhere [10], identification of scenes was more difficult in documentary and news programs, where there were sometimes extended interviews. Here, we adopted a more conservative approach so that a five minute uninterrupted interview counted as one scene.

We recorded information both about programs and scenes within programs. For programs, these features included censor classification rated as G = general exhibition; PGR = parental guidance recommended; AO = adults only; and not classified. The latter rating applied to news, sports, live programming and music programming, all of which are exempt from classification [13]. Other program characteristics included type (coded as music, comedy, news/current affairs/documentary, drama/action, reality TV, and sport); frequency of screening (coded as daily, weekly, "one-off" programs, and movies); and country of origin (coded as NZ, Australia, USA, UK, and other).

Where a scene depicted alcohol imagery, we recorded the number of people intoxicated, drinking alcohol, talking about alcohol, serving alcohol and main type of alcohol featured where possible (beer, spirits/liqueurs, wine, ready-mixers). We also coded the characteristics of the two main characters depicted in the scene including gender, an estimate of age (< 18 years, 18–29 years, 30–39 years and 40 plus years), ethnicity (European-Caucasian, Maori-tangata whenua or indigenous people of NZ, Pacific, African-American, Hispanic, Asian, Indian, and other), character's role (central character, support, extra, interviewer, interviewee, voice-over, and other), and their behavior. We coded talk about the positive or negative effects of alcohol; talk of quitting or cutting down on use of alcohol; the depiction of positive effects of alcohol (e.g. people having a good time, a party); the depiction of negative effects (e.g. drink driving, injuries, fights, addiction); and other alcohol imagery (e.g. containers, glasses, signs). All advertisements and trailers for other programs were viewed and coded for alcohol imagery including promotional advertisements for alcohol and public health advertisements cautioning about problematic alcohol use. Copies of the coding forms are available on request.

The primary coding was carried out by JK, but over half of all programs were coded by JK and RM to provide consensus agreement in coding. Tapes were coded for all tobacco, alcohol, and legal and illicit other drug use. Consequently, there were multiple viewings by JK and RM of each program, scenes within programs, trailers and advertisements. We also used free text to describe the content of scenes in detail including verbatim quotes of conversations around alcohol. Furthermore, relevant scenes were viewed to reach agreement where there were uncertainties, and coding was monitored throughout the process.

## Results

### Scenes with alcohol imagery

There were 120 programs shown over the week's viewing. Seventy-five programs (63%) had at least one scene containing alcohol imagery; 22 of these programs (18% of the total) had 10 or more scenes. There were 473 scenes with any alcohol imagery in the scheduled programs, with the maximum number being 21 in one episode of *Coronation St*. A substantial part of this UK drama series revolves around the *Rover's Return *a pub (hotel or bar). The average number scenes across the 120 programs was 3.9 (standard deviation = 5.4).

Of the 473 scenes with alcohol imagery, at least one person was drinking alcohol in 184 of them (39%). In 129 of the scenes (27%) at least one person was talking about alcohol or alcohol related topics, and in 52 scenes (11%) someone was shown serving alcohol. Only 18 scenes (4%) showed a person rated as obviously intoxicated. It should be noted that these percentages are not independent as some scenes included more than one of these behaviors and usually included other alcohol-related imagery. Alcohol use was depicted in a variety of settings: 90 scenes included a bar or pub (19%), 56 showed a party (12%) and 33 scenes were set in a restaurant (7%). Bottles, glasses, cans, beer taps, trays, signs or advertising billboards, and other related imagery were present in 402 scenes (85%).

We coded the characteristics of 439 people shown in these scenes; 65% were men and 35% women. Of those shown using alcohol, only 5% were judged to be under-age by NZ law (less than 18 years); 39% were estimated to be between 18 and 29 years, and 29% between 30 and 39 years; 23% were judged to be over the age of 40 years; and for 4%, age was too difficult to determine. Most (66%) were judged to be European-Caucasian, while 24% were judged to be African-American, all in US programs. There were relatively few individuals judged to be Maori (n = 6) or Pacific (n = 3). Of the 248 characters shown using alcohol in drama/action/comedy shows, 80% were "support" or "extras".

By and large, the imagery was judged to be "neutral," being neither directly critical of, nor directly promoting alcohol use. Some 62 scenes (13%) were rated as showing the positive effects of alcohol in terms of people obviously enjoying themselves or having a good time; e.g. a winning yachtsman shown spraying champagne over a cheering crowd. Several scenes also contained "positive" conversations about alcohol; e.g. an interviewee described how people who "are a bit more inhibited" should have one or two gins and "then go gardening and loosen up." Only 36 of the 473 scenes (8%) had imagery rated critical of alcohol use. These included comments relating to injuries, fights, mental health issues or drink-driving; e.g. a character in a drama referred to making it through the day with a "massive hangover." Only 14 of these 36 scenes contained visual imagery showing negative effects of alcohol use; e.g. a male character in a comedy was shown intoxicated, slurring his words and stumbling. Talk of quitting or cutting down alcohol use occurred in only 2 scenes.

### Type of program and alcohol imagery

Table 1 shows the extent of alcohol imagery according to type of program. The 38 music programs showed 253 music videos, sometimes combined with interviews with the musicians; 28 of these videos (11%) contained a total of 96 scenes with alcohol imagery. We examined the association between the characteristics of the programs and number of scenes with alcohol imagery using a series of Kruskal-Wallis "analysis of variance" by ranks tests for 3 or more groups [14]. This test provides a chi-square summary statistic.

**Table 1:** Alcohol imagery according to type of prime-time programme – 2004.

**Type of Programme**	Number of programmes	Programmes with any imagery (%)	Number of scenes with any imagery	Scenes with critical imagery (%)	Mean scenes per hour
**Music**	38	47.4	109	4.6	3.7
**Comedy**	17	64.7	53	17.0	5.9
News* Current affairs Documentary	32	62.5	126	4.0	4.5
**Drama action**	21	85.7	114	6.1	6.0
**Reality tv**	10	70.0	52	19.2	5.5
**Sport**	2	50.0	19	0.0	6.3
**Total**	**120**	**63.0**	**473**	**7.6**	**4.8**

* Includes sports news; 75% of the alcohol imagery in the News, current affairs and documentary category was shown in sports news items.

There was no overall significant difference in number of scenes with alcohol imagery according to type of program as shown in Table 1, chi-square (5 df) = 5.28, P = 0.383. Similarly, number of scenes did not differ significantly according to country of origin of the program [chi-square (4 df) = 4.30, P = 0.367], or frequency of screening [chi-square (3 df) = 2.79, P = 0.426]. Number of scenes, however, did differ significantly according to censorship rating [chi-square (3 df) = 11.52, P = 0.009], with fewer total scenes containing alcohol imagery in programs rated G (mean = 1.7; sd = 3.2) compared with all other programs (mean = 4.4; sd = 5.6).

### Alcohol imagery in advertisements and trailers

A total of 2581 advertisements were shown over the week's viewing; 19 (< 1%) promoted alcohol brands or retail outlets. These included a very elaborate storyline advertisement for *Stella Artois*, a Belgian beer brewed under licence in NZ, as well as advertisements for local NZ beers. A further six "community service" advertisements cautioned against excessive alcohol use (e.g. "*Don't drink and fry*," a message from the NZ Fire Service highlighting the dangers of excessive alcohol use and fires in the home), and another 99 (4% of all other advertisements) contained alcohol imagery to help sell products from soup, airline tickets, and a bank, to clothing and faster internet connections. For example, in an advertisement for a brand of building insulation, two couples were shown drinking wine in an obviously warm living room. In a community service advertisement for *Me Mutu Quit *(the national phone based smoking cessation service) in which real people tell their own stories about quitting, one character says to another "I had a bet with my mate that I'll stop smoking. He betted (*sic*) me 3 dozen that I'll be smoking by the end of the week." While not specifically mentioning beer, it is implicit in colloquial NZ usage that a "dozen" refers to beer.

There was a significant association between type of program and the number of advertisements with positive/neutral alcohol imagery [chi-square (5 df) = 24.99, P = 0.0001]. There were more such advertisements shown during drama/action (mean = 1.4 sd = 1.4) and reality TV shows (mean = 2.3 sd = 1.5) compared with other programs (mean = 0.5 sd = 0.9). Finally, there were 875 trailers containing 51 scenes with alcohol imagery; none were critical of alcohol use.

## Discussion

If we add the 473 scenes in prime-time programs, and all advertisements and trailers showing alcohol imagery, there were 648 separate depictions of alcohol imagery across the week. This amounts to an average of one depiction of alcohol imagery every 9 minutes. Scenes with neutral or positive imagery outnumbered scenes with adverse health consequences of drinking by 12 to 1. This absence of critical imagery implies few health consequences from the use of alcohol. By contrast, three-quarters of all scenes showing illicit drug use depicted adverse health and social effects [10]. Nearly two-thirds of all programs contained at least one scene with alcohol imagery. In comedies and dramas, it was typically a male support or extra, 18–39 years old, drinking alcohol. For every advertisement cautioning against excessive use of alcohol, there were 3 advertisements for alcohol products, and 16 for non-alcohol goods and services, which employed alcohol as part of the sales pitch. The CEO of one large NZ advertising agency is on record as saying that "many of the best ads in this country have been beer ads" [15]. As noted in previous research [5], advertisements promoting beer were heavily targeted at young men, and used sophisticated imagery and storylines. While advertisements marketing alcohol products may only be screened from 8.30 pm [16], others containing alcohol imagery were shown earlier.

Overall, we believe that the evidence points to a pervasive amount of alcohol imagery on NZ television. Furthermore, while there is concern about the amount of imagery relating to alcohol advertising, most of the imagery in prime-time viewing comes from other sources. Based on surveys of NZ adolescents' television viewing [17,18], the average 13 to 15 year-old watches between 3 to 4 hours per day, 7 days a week. Over one week, this may amount to viewing between 140 to 190 depictions of alcohol imagery, by and large neutral or uncritical of alcohol use. It was somewhat heartening that programs in our survey rated G contained less alcohol imagery than other shows. Still, NZ evidence suggests that children's viewing hours are very similar to adult viewing hours, and many children watch TV until 10.00 pm or after [18]. Children as young as 5 years will be exposed to a lot of alcohol imagery.

Recent research points to the effects of alcohol advertising on adolescent consumption [6-9]. What is less clear is how to counteract the adverse aspects of such advertising on adolescent behavior [19]. One potential area of action concerns strengthening the role for health professionals in educating parents about the impact of media on children, and encouraging household "rules" about media use such as reducing TV viewing [20]. At a practical health promotion level, media education programs in the school curriculum may help teach adolescents to be more "media savvy" to help counterbalance the degree of uncritical depiction of alcohol use. There is clearly a role for health professionals in advocating for reduced alcohol imagery on TV, in the same way that health professionals have advocated for industry and government action to reduce marketing of high fat, high sugar foods on children's TV [18]. For example, the New Zealand Drug Foundation has consistently advocated for the elimination of alcohol advertising on TV and radio [21]. Our study suggests that for each community service advertisement highlighting the possible adverse effects of alcohol, there were 19 other advertisements with neutral or pro-alcohol imagery. This suggests a greater role for counter-advertising on TV as part of a broader public information campaign on the risks associated with excessive use of alcohol.

## Authors' contributions

RM, JK and AR were named principal investigators on the grant for this research. All three authors jointly planned the research design and methods. The primary coding of the data was carried out by JK, with additional help and consultation from RM. The analysis was the prime responsibility of RM and JK. All three authors contributed equally to the final manuscript.
